# Feasibility and acceptability of the cross-national multisectoral OPTIM-PARK intervention for people affected with Parkinson’s disease and their family carers

**DOI:** 10.1186/s12913-026-14912-5

**Published:** 2026-06-23

**Authors:** Ellen Gabrielsen Hjelle, Tove Lise Nielsen, Line Kildal Bragstad, Naja Benigna Kruse, Louise Buus Vester, Silje Bjørnsen Haavaag, Jacob Callesen, Anita Haahr, Maria Victoria Navarta-Sánchez, Leire Ambrosio, Azucena Pedraz-Marcos, Dorit Kunkel, Ana Palmar-Santos, Lydia Lopez-Manzanares, Eline Aas, Mari Carmen Portillo

**Affiliations:** 1 Department of Public Health and Interdisciplinary Health Science and CHARM – Research Centre for Habilitation and Rehabilitation Models & Services, University of Oslo, Oslo, Norway; 2 Department of Rehabilitation Science and Health Technology, Oslo Metropolitan University, Oslo, Norway; 3 Research Centre for Prevention and Rehabilitation, VIA University College, Aarhus, Denmark; 4 Department of Occupational Therapy in Aarhus, VIA University College, Aarhus, Denmark; 5 Department of Nursing, VIA University College, Randers, Denmark; 6 Department of Public Health and Interdisciplinary Health Sciences, University of Oslo, Oslo, Norway; 7 Department of Physiotherapy, VIA University College, Aarhus, Denmark; 8 Department of Public Health - Department of Science in Nursing, Aarhus University, Aarhus, Denmark; 9 Nursing Department, Faculty of Medicine, Universidad Autónoma de Madrid, Madrid, Spain; 10 The Carlos III Health Institute (ISCIII), Madrid, Spain; 11 NIHR Applied Research Collaboration Wessex, School of Health Sciences, University of Southampton, Southampton, UK; 12 Movement Disorders Unit, Neurology Department. University Hospital La Princesa, Madrid, Spain; 13 Department of Health Management and Health Economics, University of Oslo, Oslo, Norway; 14 Division of Health Services, Norwegian Institute of Public Health, Oslo, Norway

**Keywords:** Cross-country evaluation, Family caregivers, Feasibility study, Multisectoral action, Parkinson’s disease, Parkinson’s disease coordinator, Patient involvement, Person-centered care

## Abstract

**Background:**

Parkinson’s disease (PD) is a common neurological disease worldwide. Management of PD is complex, requiring person-centred care to address the physical, emotional, social, and economic impacts of the disease. The OPTIM-PARK intervention was developed to enhance the process of living with PD for people affected and their family carers (FCs) by designing inter- and multisectoral collaboration and incorporating a coordinator role in four European countries: Denmark, Norway, Spain, and the United Kingdom. The aim of this cross-national study was to evaluate the feasibility and acceptability of the OPTIM-PARK intervention in community settings.

**Methods:**

A mixed-methods convergent design was applied, including quantitative analysis of registrations, outcome measures and an Acceptability scale, as well as qualitative analysis of Logs and Mapping of Resources, and semistructured interviews. The focus of the three months intervention was a PD coordinator who undertook a personalized needs assessment and provided information and support, strengthened multi- and intersectoral collaboration, and optimized access to and use of community resources and other third sector support systems.

**Results:**

A total of 130 participants were included in the intervention, 72 people with PD and 58 of their FCs. The intervention was typically provided in three consultations. The PD coordinator focus was on individualized support through active listening in the delivery of healthcare interventions. Inclusion of FCs were highly valued. Completion of the outcome measures was feasible, 105 (81%) participants were able to complete outcomes at both time points with <5% missing data. Most participants (92%) found it easy to answer the questionnaires, the use of digital tools for data collection was found beneficial if the participant were comfortable with the digital format.

**Conclusions:**

To the best of our knowledge this is the first cross-national European study exploring the feasibility of a PD coordinator focussed support to enhance the process of living with PD. The findings suggest that the OPTIM-PARK intervention was feasible and well-received in a community setting across different countries, suggesting potential for wider application and further evaluation in a larger-scale trial.

**Supplementary Information:**

The online version contains supplementary material available at 10.1186/s12913-026-14912-5.

## Background

Parkinson’s disease (PD) is a common neurodegenerative disease associated with dopamine deficiency and characterised by both motor and non-motor deficits [1, 2]. The number of people diagnosed with PD has been growing globally and PD is currently the fastest growing neurological disease worldwide [3].

Clinical diagnosis is based primarily on motor symptoms, but non-motor autonomic dysfunctions, such as sleep problems, mood disorders, and pain, can develop in parallel or years before a diagnosis [4]. Treatment mainly aims to reduce motor symptoms through either pharmacological interventions or advanced treatment such as deep brain stimulation [2]. Supportive treatment may consist of physio therapy [5], speech and language therapy [6], occupational therapy [7], or psychoeducative interventions [8]. Importantly, none of the existing treatments can currently cure or stop the disease. Cost-of-illness analysis has shown that costs of PD are high, mainly due to drugs, hospitalization and productivity loss, and tend to increase as the disease progresses [9]. The challenges and costs associated with living with and managing the impact of PD are considerable [2, 9], and the later stages of the disease present extensive physical and psychological impact to those affected [10, 11] and their families [12, 13].

The complexity of PD requires a person-centred approach that takes into account the stage of the disease and the management of the physical, emotional, social, and economic aspects of living with PD [14]. According to WHO Europe [15], a multi- and intersectoral action for improved health and wellbeing should be built on partnerships and coordination of services encouraging collaboration both horizontally across sectors and vertically through the levels of governance. An integrated approach with multi- and intersectoral coordination is also desired by people with PD (PwPD) and their family carers (FCs) to improve the management of PD [16], and an intervention that features communication and collaboration amongst healthcare professionals, PwPD, and their FCs is suggested to promote success [17, 18]. To enable a person-centred approach in a multi- and intersectoral collaboration, it is advisable to include professional PD support that can ensure continuous direction to available resources and to enhance multisectoral communication, such as a coordinator [19].

Research suggests that a stronger collaboration between different care levels, personalized health care according to needs, and communication between healthcare providers are crucial components to facilitate a more integrated care pathway and to promote better living with PD [18, 20]. Such an approach can lead to more targeted assessment of the individuals’ specific needs and circumstances, thereby promoting more effective self-management. Self-management programs for long-term conditions are evolving and may include collective cross-sectoral initiatives, personal networks, and various community resources [21].

Integrated action plans of multi- and intersectoral collaboration play a crucial role in reaching all areas and populations and to ensure equal access to healthcare services, particularly for those individuals living in disadvantaged areas [22, 23]. By collaborating, health and social services can more effectively develop and implement care coordination across multiple sectors for conditions such as PD. In Denmark, Norway, Spain, and the United Kingdom (UK), efforts to explore these integrated services have shown promise for the development of a care pathway for PD management [23].

Based on previous research and WHO recommendations [15–24], the OPTIM-PARK project (Optimising Community Resources and Support to Enhance the Process of Living With Parkinson’s Disease) was developed with a cross-national perspective in: Denmark, Norway, Spain, and the UK [16–18, 23]. The aim of the project was to enhance the process of living with PD for PwPD and their FCs by designing and evaluating a multisectoral intervention, including a coordinator, to optimize the use of available resources in European countries.

To the best of our knowledge, this is the first study to propose an intervention that enables full use of all public, private, and voluntary sector resources and the availability of a coordinator for PwPD and FCs. The components of the OPTIM-PARK intervention and evaluation design were evaluated to guide further development and refinement of the intervention, investigate its feasibility and acceptability as part of a possible new care pathway, and draw sound conclusions on which to plan a larger-scale evaluation.

### Aim

The objective of this study was to evaluate the feasibility and acceptability of the OPTIM-PARK intervention in a cross-country, community setting.

## Methods

### Design

This feasibility study adopted a mixed methods convergent design and included collection of quantitative and qualitative data [25]. To enhance rigour and consistency across countries the UK Medical Research Council framework for complex interventions was used in the development and feasibility testing of the intervention [26, 27]. In the current study, this involved evaluation of the feasibility of recruitment and retention, implementation of the intervention, acceptability of the intervention’s structure and content, participant responsiveness, and data collection strategies. The evaluation also incorporated cross national adaptations of all elements in the feasibility, service utilization and cost evaluation.

The manuscript conforms with the Consolidated Standards of Reporting Trials (CONSORT) 2010 guideline extension for randomised pilot and feasibility trials [28]. The project was conducted in close collaboration with patient and public involvement (PPI) groups and included multiple discussion meetings throughout the project period ranging from the planning of data collection, the development of the intervention, to the interpretation of the findings. The groups met at least six times, with meetings primarily organized at the national level. A final summary discussion was conducted digitally with participants from all countries after the interventions and data collection were finalized.

### Setting

The study was conducted in community settings in Denmark, Norway, Spain, and the UK. All recruited PwPD continued to receive usual care according to country-specific standard practices. Typically, usual care in the community consisted of treatment of medical issues by a general practitioner (GP) or neurologist in addition to nursing and therapy input and participation in relevant activities based on needs and availability in each community and country.

A PD Coordinator has not been standard practice across the country in any of the participating countries. Yet some initiatives exist. For example, some municipalities in Denmark have established a PD coordinator to support PwPD, assess their needs and to coordinate follow-up care. In 2022 and 2023, Norway introduced the ParkinsonNet model [29], in which a network of specialized healthcare professionals in the community provides high-quality care through interdisciplinary collaboration and coordination to support PwPD. Spain has a multidisciplinary approach to PD care, however mainly carried out at the specialized care level. In the UK, Parkinson nurses are located either in the community or at hospitals, with the purpose of providing expert care to PwPD.

### Recruitment of participants

Each country planned to recruit PwPD and FCs consecutively at least three months within the recruitment period from March until September 2022. All PwPD in the recruitment area and their FCs, if the PwPD agreed to include them, were considered eligible for the feasibility. Patients who were not able to provide informed consent due to cognitive impairment were excluded. Specific recruitment strategies were designed in each country based on the local conditions. In Denmark, the participants were recruited from two hospitals, two neurological clinics, and three municipalities. Norwegian recruitment took place in two municipalities via the OPTIM-PARK coordinators.

Norway experienced challenges with recruiting coordinators and failed in establishing collaboration with coordinators in time to recruit for three months within the available timeframe. Consequently, in Norway, the recruitment period lasted only one month (September 2022). Due to the time limitations of the project, expanding the recruitment period for Norway, was not possible. In Spain, participants were approached at a hospital when the PwPD arrived for appointments. The UK recruited from five Parkinson’s UK branches and two primary healthcare centres.

A sub-sample of PwPD and FCs who completed the intervention, were invited to participate in qualitative interviews after completing the intervention. A purposive sampling strategy [30] was applied to include a variety from the included participants based on factors that varied in the sample as gender, age, and time since diagnosis. The goal was to conduct interviews with five PwPD and five FCs from each participating country. This was to ensure a sufficient sample for achieving saturation [30]. Additionally, healthcare professionals from all participating municipalities centres were included in the interview process.

### The intervention

The OPTIM-PARK intervention focussed on a personalized needs assessment and provision of a single point of contact in the form of a PD coordinator, who was trained by the OPTIM-PARK research groups in each country in the intervention according to intervention material developed by the research group. In addition, follow up was according to needs throughout the intervention period. The coordinators were experienced with PwPD and were either registered nurses, nurse assistants, occupational therapists or physiotherapists. Training across all countries included an introduction to the project, clarification of its aims and purposes, and a walkthrough of all documents to be used. This was typically completed in approximately two hours and delivered either face to face or digitally, depending on preference and practicality. If necessary, coordinators received additional guidance following the initial training, along with follow-up support as needed throughout the project period.

**Fig. 1:**
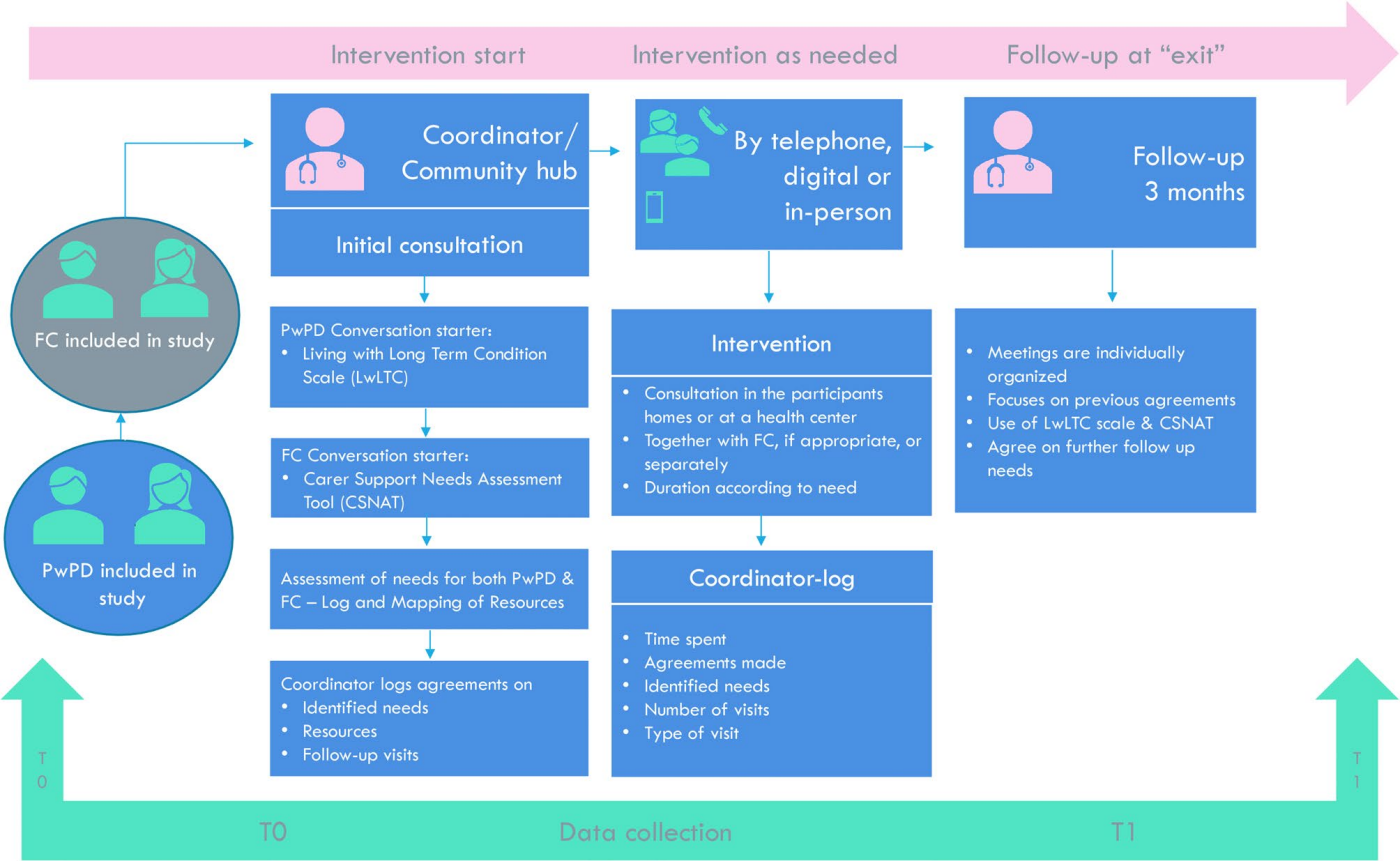
Framework of the intervention. PwPD: People with Parkinson’s disease. FC: Family carer. CSNAT: Carer Support Needs Assessment Tool. LwLTC: Living with a Long Term Condition scale

The intervention started after the inclusion of a PwPD and lasted three months. The coordinators provided personalized information and support and facilitated access to multi- and intersectoral resources as well as supporting access to and the use of community resources and private support systems for the PwPD. The consultations took place face-to-face, via videoconference, or over the telephone, depending on participant preference. All participants had at minimum, one initial meeting upon ‘entering’ the study and a follow-up meeting three months later upon ‘exiting’ it. All participants had the opportunity to schedule additional follow-up meetings during the three-month period as needed. The intervention meetings were held with the PwPD, the FCs, or the dyads, according to participant preference. A framework of the intervention process is provided in Fig. 1.

In addition to a Log and Mapping of Resources document (Supplementary file I) to keep track of appointments, meetings, and agreements, two standardised instruments were available for coordinators to use during the intervention to undertake a needs assessment as conversation starters: the Living with a Long Term Condition (LwLTC) scale for the PwPD [31–33] and the Carer Support Needs Assessment Tool (CSNAT) for the FCs [34]. While these instruments were offered as conversation tools to guide the coordinators through the first meeting, the decision of whether or how they would be applied was left up to the coordinators.

### Data collection

**Fig. 2:**
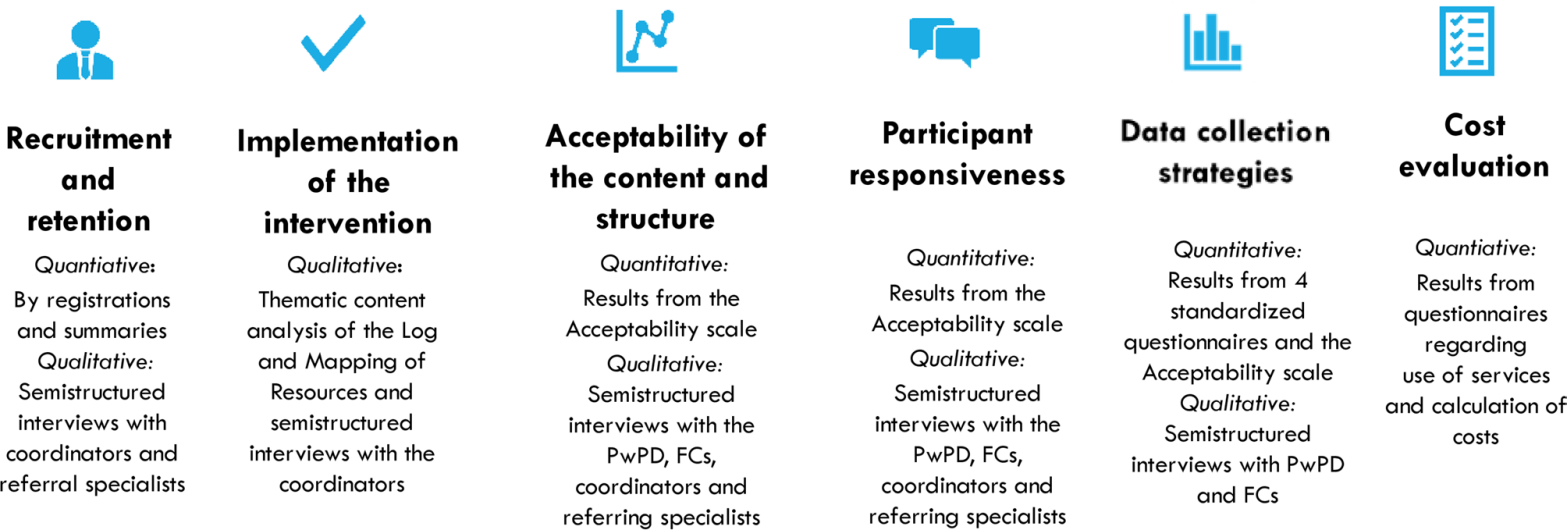
Overview of the types of data collected related to the component evaluated. PwPD: People with Parkinson’s disease. FCs: Family carers

The data collection followed a rigorous process and incorporated quantitative registrations, outcome questionnaires (see Table 1), Log and Mapping of Resources worksheets, as well as semi-structured qualitative interviews. Figure 2 offers a comprehensive overview of the types of data which informed the different components of the evaluation. In line with a convergent design [35], qualitative and quantitative data were collected and analyzed within a similar timeframe. Both quantitative and qualitative data may be collected concurrently or following each other within this timeframe. Typically, in a convergent design, the two types of data are analyzed separately before being merged [35]. In this study, an interactive approach was adopted where the data collection and analysis were iterative, thereby influencing changes in data collection procedures. For instance, initial quantitative findings influenced the types of questions and focus of the qualitative interviews.

**Table 1:** Overview of outcome measures

Concept	Scale	People with Parkinson’s Disease	Family Carers	Number of questions
Quality of life	Parkinson’s Disease Questionnaire (PDQ-39)	X		39
	PDQ-Carer		X	29
	EQ-5D-5 L	X	X	6
Social support	Duke-UNC Functional Social Support Questionnaire (FSSQ)	X	X	8
Caregiver burden	Short Burden Scale for Family Caregivers-BSFC-s		X	10

### Quantitative data

The quantitative data were collected through a socio-demographic form, standardized outcome measures, an acceptability scale developed for the project, and reports on the use of healthcare services. The questionnaires were distributed electronically using Survey Exact [36] in Denmark and the PGP encrypted Nettskjema.no [37] in Norway, while the Spanish and UK participants used paper versions. In Denmark and Norway, digital links were provided via email or SMS depending on participant preference. In Spain, the participants were scheduled for a consultation at the recruiting centre to fill out the questionnaire. In the UK, participants completed the questionnaire at home and returned it to the coordinator or a research team member either in-person or by post.

A questionnaire addressing the acceptability of several elements of participation, “the OPTIM-PARK acceptability scale”, was distributed to all participants after they finished the intervention. Danish and Norwegian participants accessed the scale through a link sent by email or SMS, while Spanish and UK participants received it by post, or filled it in by phone or in person.

Data from all countries were anonymized and uploaded for analysis via a secure data transfer link to the Service for Sensitive Data (TSD) in Norway [38], which is designed for the storage and processing of sensitive data in compliance with the Norwegian Data Protection Authority [39] and Norway’s Health Research Act [40].

#### Outcome measures

Three outcome measures for PwPD and four for FCs were used (see Table 1). Each group completed 53 items at baseline (T0) and three-month follow-up (T1). The validated [41] Parkinson’s Disease Questionnaire (PDQ-39) for PwPD has 39 items across eight domains: mobility, ADLs, emotional wellbeing, stigma, social support, cognitions, communications, and bodily discomfort. The Parkinson’s Disease Questionnaire-Carer (PDQ-Carer) [41] has 29 items in four domains: social and personal activities, anxiety and depression, self-care, and stress. Both use a five-point scale (0 = never to 4 = always).

The Duke-UNC Functional Social Support Questionnaire (FSSQ) [42] The Duke-UNC Functional Social Support Questionnaire (FSSQ) includes eight items on social support, rated on a five-point Likert scale (1 = much less support than desired to 5 = as much support as desired).

The 5-level EQ-5D version, EQ-5D-5L [43], a generic questionnaire validated for a PD population [44] measures health-related quality of life across five dimensions: mobility, self-care, usual activities, pain and discomfort, and anxiety and depression. Each dimension has five levels (1 = no problems to 5 = extreme problems/unable). It also includes a visual analogue scale (VAS) from 0 (worst imaginable health) to 100 (best imaginable health). The short version of the Burden Scale for Family Caregivers (BSFC-s) [45] consists of 10 items on caregiving burden, rated on a four-point Likert scale (0 = strongly agree to 3 = strongly disagree). A sum score (0–30) below 20 indicates burden [45].

#### The OPTIM-PARK acceptability scale

The research team created the OPTIM-PARK acceptability scale in English and translated it into Danish, Norwegian, and Spanish. The scale consists of 45 items in six categories: participation, content, intervention format, collaboration with the coordinator, perceived usefulness, and questionnaire acceptability. Responses are on a four-point Likert scale (0 = strongly disagree to 3 = strongly agree) with an additional ‘not applicable’ option and space for free-text comments.

In the UK, a preliminary version of the OPTIM-PARK acceptability scale with 31 items across five categories was used: participation, intervention content, coordinator collaboration, perceived usefulness, and questionnaire acceptability. This version used a five-point Likert scale (0 = strongly disagree to 4 = strongly agree) with a neutral middle category. There were 28 overlapping items between the two versions of the scale, which were included in the cross-country analysis. Non-overlapping items were reported separately.

#### Registration of the use of healthcare services

Healthcare service use was recorded at baseline and follow-up, including visits to GPs, physiotherapists, occupational therapists, speech and language therapists, home care nursing, home care support, neurologists, and hospital admissions. Participants also reported the duration and frequency of informal care and practical support from FCs. This data was used to calculate resource use for each participant over the three months of the OPTIM-PARK intervention.

The cost of the intervention was estimated using UK National Health Services tariffs [46]. The unit cost of a physiotherapist was used to approximate coordinator costs for cross-country comparison, regardless of their professional background. Informal care costs were estimated based on an hourly wage. Total costs per participant over the three months were estimated by summarizing the costs of the coordinator, healthcare services, and informal care.

### Qualitative data

Qualitative data collection included the coordinator’s log data and individual semi-structured interviews with PwPD, FCs, referring specialists, and coordinators. Three slightly different interview guides were developed in English, Danish and Norwegian by the Danish and Norwegian teams and translated into Spanish: one for PwPD, one for FCs, and one for coordinators and referring specialists. The interview guide is provided in supplementary file II.

#### The log and mapping of resources

To ensure implementation fidelity, each country coordinator was given a “Log and Mapping of Resources” template (Supplementary Material I) to guide participant meetings. Developed by the Danish and Norwegian teams in English and translated into Danish, Norwegian, and Spanish, this document recorded the date, duration, and agenda of each meeting. The template listed 28 resources classified as beneficial to PwPD and/or FCs based on previous work [23], organized into “Body function and body structure,” “Activity and participation,” and “Environmental factors.” Each country team identified local resources fitting these classifications, creating community resource maps that included supports for living with PD from multiple sectors.

The coordinator personalized resource recommendations to address individual participants’ needs, checking if these resources were known, needed, requested, or used. The goal was to document the coordinator’s actions, such as providing referrals, making contacts, or supplying information. At follow-up and final meetings, these logs were used to track changes and monitor agreements and arrangements. After the intervention period, the log notes were collected based on the coordinators consent to share them. Each country’s research team then summarized these notes in English according to a template developed by the research team. An example of this template is provided in Supplementary file III. All the summaries (one per participant or dyad, *n* = 58) were uploaded to TSD for further analysis.

#### Semi-structured qualitative interviews

The main topics for the qualitative interviews with PwPD, FCs and healthcare professionals were acceptability of the intervention, participant responsiveness, the delivery of the intervention, contextual factors, and acceptability of the outcome measures.

## Analysis

### Quantitative analysis

For the quantitative analysis, samples from all four countries were combined into a unified European sample. Stata Statistical Software [47] was used to analyse outcome measurements, including descriptive statistics (frequency, means (SD), median (min-max)), and examination of normality plots and distribution.

SPSS Statistics for Windows, Version 29.0 [47] was employed for the Acceptability scale analysis. Recoding of the grading scale for the UK sample was performed to permit analysis of the 28 overlapping items collectively across countries. All items were analysed descriptively, with frequencies and cross-tabulations indicating the proportion of the sample based on a dichotomization of the Likert scale reflecting agreement versus disagreement with statements.

### Qualitative analysis

The main objectives of the qualitative part of the current study were to identify cross-country similarities and enable comparison with the quantitative findings. A total of 58 Logs and Mapping of Resources documents were obtained, summarized in English, coded and analysed by the Danish and Norwegian teams. The basic thematic content analysis described by Braun and Clarke [48] was applied with a mix of inductive and deductive approaches. All topics were organized in an Excel spreadsheet and analysed to ascertain frequently addressed topics and determine the main focus of the delivered intervention. During this process, the findings were iteratively discussed with all authors to reach consensus about which topics to pursue. The themes were then divided into four categories (Fig. 4). The framework for organising the level of focus was Anne Fisher’s [49] model on intervention types, which suggests focusing either on maintaining function or on restorative or adaptive approaches.

**Fig. 3:**
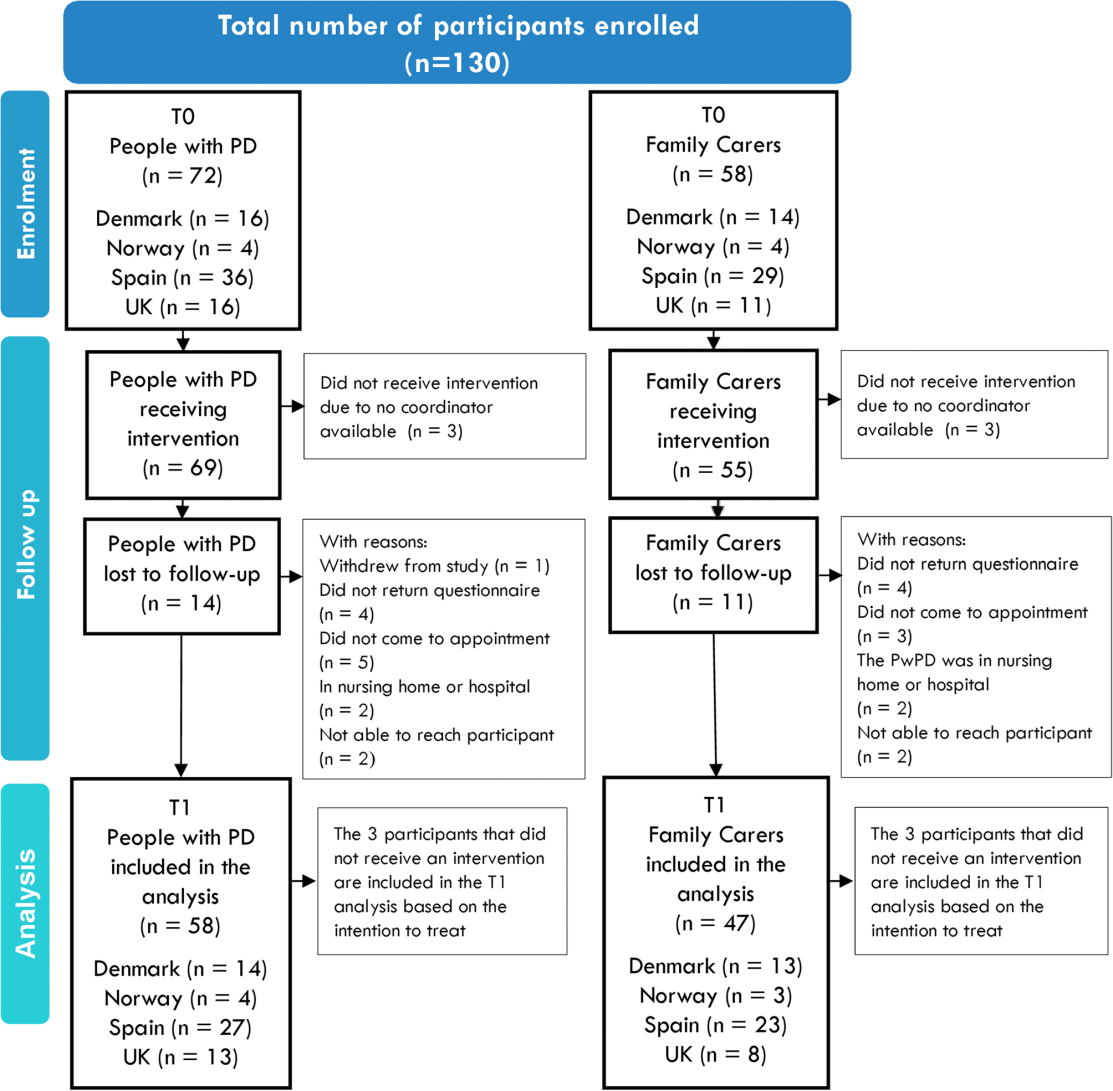
Flow chart

**Fig. 4:**
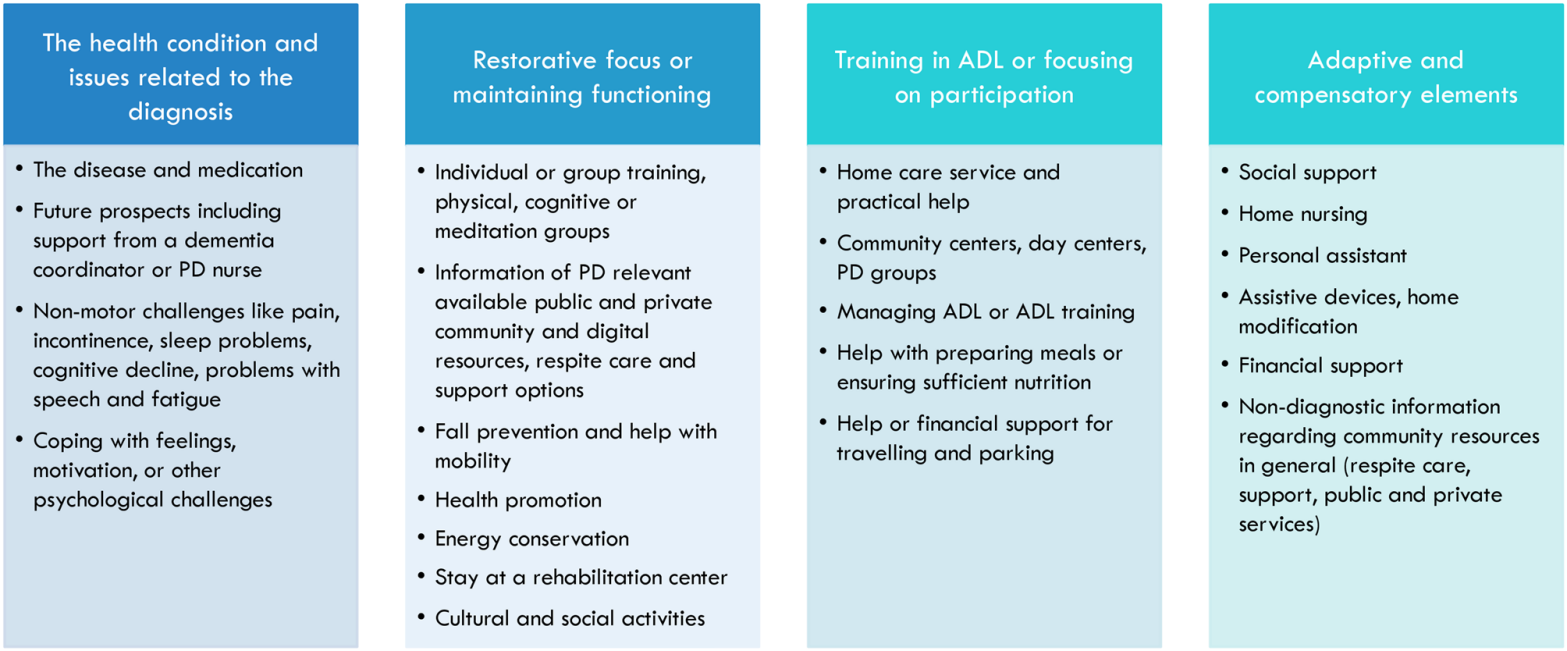
Main topics of the intervention

The qualitative interviews were summarised by each country’s research team following a predefined template, focussing on data addressing the acceptability, feasibility and meaningfulness of the intervention developed by members of the Danish team with an inductive approach to the data.

## Results

The results are organised according to the five elements evaluated in this feasibility and acceptability study: recruitment and retention, implementation of the intervention, acceptability of the content and structure, participant responsiveness, the data collection strategies and cost evaluation.

### Recruitment and retention

A total of 130 participants (72 PwPD, 58 FCs) were included in this study. Of the 151 PwPD and FCs, who were eligible in the three countries where data on eligibility were available (Norway, Spain, and the UK), 21 declined. The most common reason for declining was being “too busy” (*n* = 9). Other reasons were “too much information or intrusive questions” (*n* = 2), “lack of interest” (*n* = 2), and “living too far away” (*n* = 2). Some individuals reported “no specific reason” (*n* = 7).

Twenty-five participants dropped out between the baseline and follow up assessment (*n* = 14 PwPD, *n* = 11 FCs). The main reason for dropout at baseline was that the participant did not attend the appointment to complete the questionnaire (in Spain) or did not return the questionnaire (in the UK). Figure 3 identifies the flow chart of the recruitment process and country specific details of the participants as well as reasons for dropout.

Of the 105 participants who completed the intervention and follow-up at three months, 78 (74%) answered the Acceptability scale questionnaire. The sample for this questionnaire consisted of 27 participants from Denmark (*n* = 14 PwPD, *n* = 13 FCs), seven from Norway (*n* = 4 PwPD, *n* = 3 FCs), 30 from Spain (*n* = 16 PwPD, *n* = 14 FCs), and 14 from the UK (*n* = 9 PwPD, *n* = 5 FCs).

Nineteen PwPD, 16 FCs, and 16 healthcare professionals (13 OPTIM-PARK coordinators and three referring specialists) were interviewed about the process and acceptability of the intervention.

The mean age of the PwPD was 75 years (95% CI: 73–77 years). While some had been living with PD for as long as 27 years, the mean disease duration was 8.1 years. A total of 78% lived with a partner, and only 14% lived completely alone. Higher education was reported by 74%, and most of the participants (83%) were retired. More than half received daily help from an informal FC. Most FCs were spouses or partners, and 57% were women. Amongst the FCs, 38% had been diagnosed with a health condition themselves. A total of 60% of the FCs were retired, and 38% shared the informal care responsibility with someone else. Table 2 provides more details. Country-specific information may be made available upon reasonable request to the corresponding author.

**Table 2:** Baseline sociodemographic data of the cross-national sample of PwPD and FCs

People with Parkinson’s disease	n = 72
Women	26 (36%)
Age	75 (95% CI: 73–77)
Years with PD diagnosis	8.1 (95% CI: 6.7–9.5) (min:1, max: 27)
Comorbidity (*n* = 70)	46 (65%)
Married or living with a partner	56 (78%)
Education (*n* = 68)	
– None	5 (8%)
– Elementary/primary school (up to 10 years)	8 (12%)
– High school (up to 13 years)	4 (6%)
– Highest level of education (min 3 years post high school)	51 (74%)
Working (*n* = 71)	
– Full or part time	7 (10%)
– Retired	59 (83%)
– Other	5 (7%)
Living area (*n* = 71)	
– Rural < 2500	5 (7%)
– Small town 2501–10000	16 (23%)
– City > 10000 (City)	50 (70%)
Living arrangement	
– Alone	10 (14%)
– With someone	62 (86%)
Receives financial aid (*n* = 71)	10 (14%)
Daily help from informal Family Carer	36 (58%)
Part of an ethnic minority	0 (0%)

Family Carers	n = 58
Women	33 (57%)
Relationship (*n* = 56)	
– Spouse/partner	43 (77%)
– Child	3 (5%)
– Sibling or Other	10 (18%)
Suffer from a disease themselves	22 (38%)
Shares the caring responsibility with someone	17 (30%)
Education	
– None	0 (0%)
– Elementary/primary school (up to 10 years)	6 (10%)
– High school (up to 13 years)	9 (16%)
– Highest level of education (min 3 years post high school)	40 (69%)
– Other	3 (5%)
Working (full or part time)	17 (30%)
– Retired	35 (60%)
– Other	6 (10%)

### Implementation of the intervention

The intervention was provided either one-on-one to PwPD or FCs, or to both as dyads. Most participants (*n* = 37, 64%) had three meetings, while ten participants (17%) had two, seven participants (12%) had four, and four participants (7%) had one meeting. Most first meetings were face-to-face (*n* = 55, 95%), with subsequent communication via telephone, in person, or videoconference. Meeting durations varied, with the first meeting lasting up to two hours and follow-up calls being shorter.

A total of 58 Log and Mapping of Resource documents (one per PwPD or dyad) were collected and analysed based on the willingness of the coordinator to share their notes. These logs structured initial conversations, tracked agreements, and assessed intervention progress. Coordinators used them in a structured manner, addressing each resource line for participants. Most intervention topics were raised by PwPD, while FCs focused on PwPD needs, providing direct help or increasing training and follow-up. Figure 4 categorizes topics by health condition, maintaining function, and restorative or adaptive approaches. The most frequently addressed topics in each theme are stated at the top in each column and include issues regarding the disease and medication, exercise and training, home care services, practical help and social support. Figure 4 presents the main topics addressed in the intervention (*n* = 58 logs)

### Acceptability of the content and structure

Findings from the Acceptability scale indicated that 79% of participants found the project personally useful. Most participants (92%) felt able to discuss their needs with the coordinator, and 93% felt encouraged to express their opinions. The majority (97%) felt listened to and taken seriously. Additionally, 91% of PwPD and 85% of FCs felt the coordinator understood their needs, and 96% trusted the coordinator to help them. A total of 92% found receiving information about available resources useful.

Qualitative interviews revealed that coordinators needed time and managerial support to stay updated on community services, which changed regularly. One coordinator noted: ‘*There’s new resources that come out all the time for patients and keeping that directory updated is going to be a real key thing, I think, for the intervention*’ (Coordinator).

After conversations with coordinators, 91% of participants felt capable of deciding which resources to pursue. Participation motivated 62% to seek other community resources. A large proportion of PwPD (89%) and FCs (81%) felt the coordinator connected them with relevant resources. However, actual utilization of these resources was lower, with 66% of PwPD and 54% of FCs using them.

### Participant responsiveness

A total of 88% of the PwPD and 78% of the FCs would recommend the project to others. A slight majority (52%) desired a longer participation period, finding three months too short, and wished for more frequent interactions with the coordinator who they appreciated was ‘*there for them’*. Additionally, 81% of PwPD and 74% of FCs gained a better understanding of PD challenges.

### The data collection strategies

According to the Acceptability scale, most participants found it easy to answer the questions at both baseline (91%) and follow-up (92%), indicating the questions were comprehensible and acceptable. Among PwPD, 86% felt they could communicate important aspects of their situation through the questionnaires, while 77% of FCs felt the same.

Most PwPD and FCs felt the outcome questionnaire questions were appropriate and not overly invasive. However, 55% found completing the questionnaires time-consuming, and 56% felt there were too many questions. Some noted question overlaps and difficulty understanding certain questions. Additionally, FCs who were not partners or spouses of PwPD felt the questions were more targeted towards partners and spouses. Baseline and follow-up results for the outcome measurements were explored. Missing data for single items was low (<5%) for 81% of participants (both PwPD and FCs) at baseline, with no completely missing datasets. Floor and ceiling effects were assessed through total score responses and normality plots. Most PDQ-39 and PDQ-Carer domains showed approximately normal distribution, with low risk of floor or ceiling effects. The EQ-5D-5L scores were normally distributed for PwPD but skewed to the right for FCs, as expected. FSSQ scores were slightly skewed to the right for both groups, indicating a potential ceiling effect. The BSFC-s was clearly skewed to the right, suggesting it may not have been sensitive enough to capture FCs’ experienced burden.

Overall, minimal positive changes were observed at the three-month follow-up in the outcome measurements. The largest change in the PDQ-39 was four points on average in the emotional well-being domain. In the EQ-5D-5L, no domain changed more than 0.1 point on average, with the VAS score for overall health increased from a mean of 57 (CI:52–62) to 61 (CI:55–66). On FSSQ question eight, the score increased from 4.2 (CI:4.1–4.7) to 4.6 (CI:4.4–4.8), with other questions changing no more than 0.2 points. Tables 1 and 2 in Supplementary file IV provide detailed results and missing data information for each outcome.

### Cost evaluation and the use of healthcare services

The intervention included at least four hours with a coordinator (2–3 contacts or visits) equal to €292 per participant. Exploring the means for the total sample at baseline and follow up, showed a modest increase in the use of three healthcare services for PwPD: physiotherapy (+14%), speech and language therapy (+11%), and follow-up with a neurologist at an outpatient clinic (+6%). The UK saw the largest increase in physiotherapy usage, rising from 19% (3 of 16) at T0 to 70% (9 of 13) at T1. In Denmark, speech and language therapy usage increased from 0% (0 of 16) at baseline to 29% (4 of 14) at T1. The questionnaire regarding use of health care services for the PwPD, also included questions about informal care and practical help. It was clear that some of the participants, particularly in Spain, received a substantial amount of help from FCs but struggled to specify the total number of hours per week. Some Spanish participants (*n* = 8) reported receiving informal care or practical help ‘24/7’, for ‘168 hours’, or ‘continuously’ at baseline, follow up or both. Table 3 lists more details about the use of healthcare services, including the costs associated with the various types.

**Table 3:** The use of public or private healthcare services for PwPD

Type of health care service Yes/No and number of visits if “Yes”	Baseline T0 n = 72	Follow-up T1 n = 58	Unit Cost Euro *
The intervention; Follow up by a Coordinator (4 hours)			€292
General practitioner	48 (67%)	38 (67%)	€39.13
Number of visits in previous three months	2 (0–6)	2 (1–10)	
Physiotherapist	29 (42%)	31 (56%)	€73.14
Number of visits in previous three months	10 (3–20)	10 (1–30)	
Occupational therapist	9 (13%)	7 (13%)	€99.11
Number of visits in previous three months	1 (0–2)	1 (0–5)	
Speech and language therapist	8 (11%)	12 (21%)	€196.55
Number of visits in previous three months	3 (2–12)	3 (0–24)	
Practical assistance from home care service	25 (35%)	16 (28%)	-
Number of visits pr week	**	**	
Home nursing care	6 (8%)	6 (11%)	€53.74
Numbers of visits pr week	20 (10–30)	15 (10-140)	
Outpatient treatment/Neurologist	41 (57%)	36 (63%)	€231.88
Number of visits in previous three months	1 (0–9)	1 (1–3)	
Dietician/nutritionist	5 (7%)	4 (7%)	€54.21
Number of visits in previous three months	4 (1–6)	3 (1–12)	
Inpatient admissions to hospital	7 (10%)	5 (9%)	€6536/€2723

Reporting in Frequency (%) or Median (min-max) accordingly

*: Unit costs are based on the tariffs from the National Health Service [46]. Costs reflect 2021£. Pragmatically, we assume that all countries have a similar unit cost for the healthcare services

**: It is not feasible to summarize a valid response, as several participants (25%) had estimated a number indicating help ‘24/7’, ‘168 hours’ or wrote out a response (e.g. ‘all the time’) for this variable

## Discussion

To the best of our knowledge this is the first cross-national European study exploring the feasibility and acceptability of a PD coordinator focussed support. The findings demonstrate that the OPTIM-PARK intervention delivered in community settings is feasible and acceptable across diverse European contexts, which brings significant progress to the field. In the following section, results concerning recruitment and retention, implementation of the intervention, acceptability of the intervention’s structure and content, participant responsiveness, data collection strategies, service utilization and cost evaluation will be interpreted and discussed, and methodological considerations will be addressed.

Recruitment strategies for this study varied between sites which reflects the study’s pragmatic approach. Some participants were recruited through the healthcare sector, while others, already familiar to the recruitment personnel, were directly contacted. In the UK especially, recruitment through third sector organizations was found more feasible, with shorter timescale. By the applied recruitment strategies, several of the recruited participants were already experienced with receiving some of the available services, including follow-up care, or were at least already aware of some of the services offered by the community and PD associations. This starting point may have influenced the referral rate to services and activities. According to previous studies, vulnerable people from ethnic minorities, older patients, and those with more medical comorbidities are typically harder to recruit [50]. While in the present study, older patients were well represented and 65% had comorbidity, we did not manage to include participants from ethnic minorities, even when the areas of inclusion were multicultural. The reason for this needs to be explored further in future studies. Another potential source of bias in our sample is the geographical location of participants, as only a small proportion (7%) lived in rural areas (<2500 inhabitants), areas that are often linked to health inequities due to limited access to healthcare services and environmental factors [51, 52]. Other concerns include that 74% reported having higher education and only 7% had no education. In sum, we cannot claim that our included sample is representative of all PwPD. Individuals from vulnerable or disadvantaged populations were likely underrepresented, and they may experience lower levels of health literacy as well as additional challenges and needs related to living with PD than those reflected in the recruited sample. Future studies should aim to employ a more comprehensive recruitment strategy to ensure the inclusion of populations from all areas and socioeconomic statuses.

The retention rate was 81%, close to the median retention rate of 88% found in a systematic literature review on clinical trials from 2021 [53]. A total of 14 PwPD and 11 FCs dropped out before T1, their exact reasons for withdrawal remain unknown. However, based on insights from our qualitative interviews, it can be speculated that some participants might have found the structured data collection process with many questions burdensome. Some may have opted out due to digital problems, especially in Denmark and Norway where digital questionnaires were used. Other factors, such as difficulties with travel or issues related to disease progression, which have been highlighted in previous research [50], may also have influenced participant dropout rates.

Concerning the implementation of the intervention, the coordinators received the planned minimum amount of training, supplemented with additional training as needed. Some variation in training across countries occurred, which was considered acceptable given the study’s pragmatic approach and the anticipated differences in expectations, needs, and cultural contexts. Intervention fidelity was examined through the Log and Mapping of Resources document, which provided insight into key factors in the lives of PwPD and their FCs. The level of detail varied due to differences in coordinators’ reporting practices and their use of the document. While the Log succeeded in capturing information across relevant areas, the logging process was perceived as somewhat complicated and time consuming. The tools accessible to support the intervention for the coordinators, the LwLTC scale and CSNAT were perceived as feasible and useful as they helped the coordinator understand the lay of the land for the participants. The scales are validated and found feasible in previous international studies when applied in a PD or FC population, either to evaluate living with the illness [54] or assess caregiver needs [55]. However, some coordinators found that filling in the forms was a long process that left little time for talking about the issues uncovered by the tools or issues that the scales did not address. For future studies, a less detailed resource mapping tool and a more pragmatic approach to the use of supplementary tools should be considered.

In addition to a lack of knowledge about PD, the most frequent issues addressed during the consultations were a need for information and options concerning exercise, home care services and social support. These services, although available through either public, private or volunteer services in all the participating countries, were seemingly not generally known among the participants. This confirmed the necessity of providing substantial information and support, as is also emphasized in the ParkinsonNet model [29], to better manage the disease-related challenges. Findings from a scoping review on multisectoral integrated care initiatives for people living with PD and their FCs [19] support the significance of components like support and the presence of a coordinator. Specifically, according to the review, the coordinator’s role was particularly important in building confidence, trust, and support; facilitating positive changes in health outcomes, and strengthening multi-agent collaboration and personalized assistance [19].

The qualitative interviews indicated that coordinators were successful in tailoring the intervention to meet individual needs, a factor likely contributing to the high retention rate. Most participants found the intervention structure acceptable and the content useful, relevant, and sufficiently individualized, which seem to meet the needs among PwPD and their family carers found in prior studies [16–18]. Over 90% of our participants reported involvement at either shared or autonomous decision-making levels; this aligns with Thompson’s taxonomy of involvement in healthcare consultations [56]. Interestingly, nearly 40% of the participants reported not utilizing available resources at all after the intervention, contrary to what one may expect to happen with increased knowledge and availability [18, 29]. Whether participants felt they did not need a service or were reluctant to use resources for other reasons were not elaborated upon, and healthcare providers may need to focus on both increased awareness and promoting utilization to optimize the use of available services.

Furthermore, the intervention’s usefulness was evident for the FCs, who expressed that their needs were commonly not addressed as part of the usual care provided in their community. Caregiver burden has been documented in previous studies [57] as has the need for FC support to reduce their burden [12, 13, 17]. The FCs advocated for the inclusion of their specific needs as caregivers to a person with PD to be a standardized part of the provided support and follow-up.

The descriptive statistics revealed only minor changes between the two time points for all outcome measurements for both the PwPD and FCs. For the PwPD, this can potentially be attributed to several factors. First, a period of three months may be too short to observe a significant change in the experience of living with a progressive chronic illness [1]. Second, irrespective of any intervention, the inevitable progression of the illness might limit the potential for noticeable improvement [1, 2]. Caution is warranted when interpreting the statistical results, as the study, being a feasibility trial, was not powered to enable robust statistical inferences.

The digital data collection in Denmark and Norway proved to be beneficial in reducing errors, such as coding responses within specific numerical ranges and preventing incorrect completion of the form and text based entries. It also minimized the risk of typographical errors and helped ensure that no questions were left unanswered. However, 52% of the PwPD, 72% of the FCs reported challenges with using online platforms in their daily life in general. This highlights the need to account for participants’ digital literacy when implementing such tools. The use of digital platforms is facilitated by modern technology, a strategy advocated in the study by Ellis and Earhart on digitally applied therapy for PwPD [58]. They recommend increased use of digital platforms for PwPD but also acknowledge the need to tailor these platforms to address the progressive motor and non-motor challenges characteristic of PD. For some participants, the digital format of questionnaires in the current may have been onerous. For future evaluations, it is recommended that data collection requirements are reduced to the bare minimum to facilitate participation and avoid multiple measures with overlapping questions

The outcome measure scorings were generally congruent with our pre-existing knowledge of appropriate scoring conventions. However, some exceptions were noted. The FSSQ scores suggested that our sample consisted of individuals with sufficient support, underlining the need to reach those without any service follow-up. Furthermore, the BSFCs, that had not previously been used in a PD population, did not meet our expectations based on knowledge of the FCs burden from earlier project phases [16, 17]. The reason for this, whether due to low instrument sensitivity or missed participants with caregiver burden, is unclear, but given that none of the participants reached the caregiver burden cut-off (11 out of 30), future studies should consider other methods to assess caregiver burden.

Regarding service utilization and cost evaluation, we observed a modest increase in the use of physiotherapy, speech and language therapy, and outpatient clinic or neurologist visits during the three-month project period. This increase may partly reflect participants’ improved awareness of available services following the intervention. However, given the progressive nature of PD [1, 4], these findings should be interpreted with caution. Symptom progression during the intervention period may independently lead to greater service utilization, making it difficult to attribute changes solely to the intervention. At the same time, the intervention encourages more extensive use of community based resources, which are generally less costly and may help prevent high cost events such as hospitalization [59]. Although such shifts in service use have the potential to reduce long-term healthcare expenditures, the integration of a coordinator into standard care will nonetheless require dedicated funding. This includes not only the coordinator’s time allocated to the intervention but also potential travel expenses and the need for ongoing follow-up across the individual’s progression of the condition.

### Limitations of the study

This study employed various tools for data collection and to guide the coordinator in conducting the intervention, and most instruments were available in all languages or translated as part of the OPTIM-PARK project. However, the Log and Mapping of Resources document required translation for use across countries. The translation process faced challenges as certain terms lacked direct equivalents in all languages, necessitating cultural translations. Cross-national discussions took place to bring the versions as close as possible across countries. For future cross-national studies using this document, a thorough forward-backward translation process and a cross-cultural adaptation is recommended to ensure linguistic and conceptual consistency. This issue also relates to cross-cultural validity more generally, as linguistic nuances and cultural meanings can vary across contexts.

A potential bias in the acceptability scale results might exist due to the use of two different versions. The UK sample used one version, while Denmark, Norway, and Spain used another due to the timing of ethical committee requirements in each country. However, the majority of the questions were overlapping and recoding of responses enabled cross national use of the results for most domains.

In terms of recruitment, the statistics suggest that the study did not successfully reach participants in rural areas or particularly vulnerable populations, such as ethnic minorities. If they were not regularly in contact with a neurologist, a Parkinson’s Association, or the health and social care systems, they may never have been approached.

## Conclusions

This cross-national study has shed light on how complex challenges are approached and how multisectoral interventions for people with PD and FCs can be developed. The OPTIM-PARK intervention was feasible and well received in community settings across countries, suggesting potential for wider application. The role of the coordinator was crucial, underscoring the importance of person-centeredness, individualization, and active listening in delivering healthcare interventions.

Addressing the needs of FCs has been an important component of the intervention. As their needs were not commonly addressed in usual care, this study highlights the necessity of incorporating caregiver specific considerations into standard care provision.

Further research is needed to refine the recruitment strategy to ensure inclusion of populations across regions and socioeconomic backgrounds. The assessments and tools applied, as well as the content of the intervention, also require refinement. It is additionally important to investigate reasons for not utilizing available resources despite awareness of them. Finally, the effectiveness of the intervention should be evaluated in larger scale trials.

## Electronic supplementary material

Below is the link to the electronic supplementary material.

Supplementary Material 1

Supplementary Material 2

Supplementary Material 3

Supplementary Material 4

## Data Availability

The datasets generated and analysed during the current study are not publicly available due to strict data protection laws in participating countries. However, anonymized datasets may be made available from the corresponding author on reasonable request.

## Abbreviations

ADL: Activities of Daily Living
BSFC: Burden Scale for Family Caregiver
CSNAT: Carer Support Needs Assessment Tools
EQ-5D-5L: The 5-level EQ-5D version
FCs: Family Carers
FSSQ: Duke–UNC Functional Social Support Questionnaire
LwLTC: Living with a Long Term Conditions
PD: Parkinson’s disease
PDQ: Parkinson’s disease Questionnaire
PDQ-39: The Parkinson’s Disease Questionnaire
PDQ-Carer: The Parkinson’s Disease Questionnaire - Carer
PwPD: People with Parkinson’s disease
VAS: Visual Analogues Scale
QoL: Quality og Life

## Declarations

### Authors’ information

Information removed for blinded review.
