# Supplementary material for: Feasibility and acceptability of the cross-national multisectoral OPTIM-PARK intervention for people affected with Parkinson’s disease and their family carers
Source: BMC Health Serv Res. 2026 Jun 23;26:999. doi: 10.1186/s12913-026-14912-5 (PMC13386678; doi:10.1186/s12913-026-14912-5)
Supplement: Supplementary file 1 — Supplementary Material 1 [file 12913_2026_14912_MOESM1_ESM.pdf]

OPTIM-PARK

**PD** ☐<br><br>**FC** ☐	**Date**<br><br>Start:<br><br>End:	**Contact person:**<br><br>**Next meeting:**

**Meeting number**	**Participants**	**Agenda including summary of the meeting and agreed follow up**	**Mode**			**Time used (minutes)**
			Phone	Digital	In person	

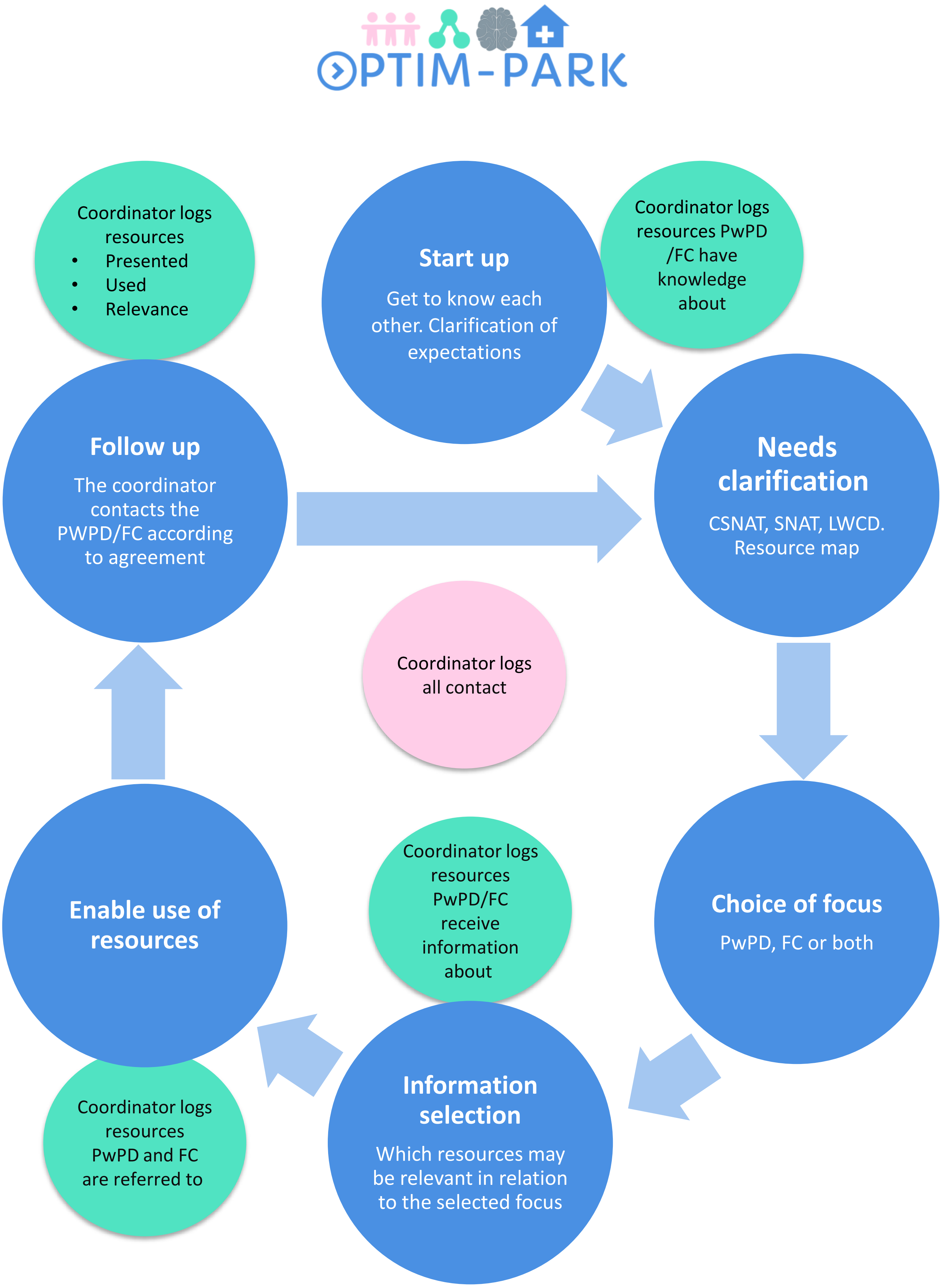
OPTIM-PARK
Coordinator logs resources
• Presented
• Used
• Relevance
Start up
Get to know each other. Clarification of expectations
Coordinator logs resources PwPD /FC have knowledge about
Follow up
The coordinator contacts the PWPD/FC according to agreement
Needs clarification
CSNAT, SNAT, LWCD. Resource map
Coordinator logs all contact
Enable use of resources
Coordinator logs resources PwPD/FC receive information about
Choice of focus
PwPD, FC or both
Coordinator logs resources PwPD and FC are referred to
Information selection
Which resources may be relevant in relation to the selected focus

OPTIM-PARK

LOG of resources, referrals and use

Public – Private – Voluntary based
Local or national

Resources that may be useful	Specify addressed resource	Aware of resource beforehand?		Expressed need?		Enabled use (e.g, referred)		Used? (comment why/why not on next page)	
		YES	NO	YES	NO	PwPD	FC	T0	T1
**Body function and body structure**									
General Practitioner									
Neurologist									
Parkinson Nurse									
Psychologist or Neuropsychologist									
Psychiatric Nurse									
Dementia coordinator									
Physical therapy									
Occupational therapy									
Speech and language therapy									
Group training (e.g. boxing)									
Other									
**Activity and participation**									
Home care nursing									
Rehabilitation Center									
Help with domestic life (ex grocery shopping)									
Personal Assistant									
Respite care									
Meetings/conversations with peer or groups of peers									
Meetings with groups of peers									
Caregiver groups									
Family welfare services									
Activities and meetings organized by Volunteers									
Other									
**Environmental factors**									
Social worker support									
Financial support									
Social security options									
Transportation aid									
Labor and employment service									
Assistive devices/technical aids									
Housing options									
Home adaption									
Other									

This is a space for further comments
