# Supplementary material for: Feasibility and acceptability of the cross-national multisectoral OPTIM-PARK intervention for people affected with Parkinson’s disease and their family carers
Source: BMC Health Serv Res. 2026 Jun 23;26:999. doi: 10.1186/s12913-026-14912-5 (PMC13386678; doi:10.1186/s12913-026-14912-5)
Supplement: Supplementary file 2 — Supplementary Material 2 [file 12913_2026_14912_MOESM2_ESM.pdf]

Interviews – feasibility and acceptability - Thematic interview guide

Acceptability of the intervention (Perceptions on acceptability; content, consultation format, time spent, perceived usefulness)

PwPD	FC	Coordinator/referring specialist
What did you think of the OPTIM-PARK project?<br>- Expectations when you joined the project<br>- Content of the project<br>- Consultation format<br>- Time and effort required to participate and adhere to the project.<br>- Would you want to continue with the project if it continues? If yes, why and if not why?<br>- How many times did you meet with the coordinator?<br>- What was the focus for these meetings?	What did you think of the OPTIM-PARK project?<br>- Expectations when you joined the project<br>- Content of the project<br>- Consultation format<br>- Time and effort required to participate and adhere to the project.<br>- Would you want to continue with the project if it continues? If yes, why and if not why?<br>- How many times did you meet with the coordinator?<br>- What was the focus for these meetings?	What did you think of the OPTIM-PARK project?<br>- Expectations when you joined the project?<br>- Content of intervention, map of resources, consultation format, time management, logging of activities<br>- Would you be happy to continue with this role/intervention?<br>- Was there anything new to your approach to the PwPD and FC, and if yes, what?
Have you experienced any benefits or challenges from participating in this project?<br>- New knowledge of resources, access to support, positive changes to life with PD, other…<br>- Too time consuming because of work or other commitments, because of disease manifestation	Have you experienced any benefits or challenges from participating in this project?<br>- New knowledge of resources, access to support, positive changes to life with PD, other…<br>- Too time consuming because of work or other commitments, health issues	What benefits and challenges do you think participating in this intervention had for PwP, FC, for yourself and other HCP?<br>- Benefits e.g.: new knowledge of resources, access to support, positive changes to life with PD, other…<br>- Challenges e.g.: time consuming or hard to integrate in my other workload
Would you recommend this project to others with PD? If yes, why and if not why?	Would you recommend this project to other FC´s? If yes, why and if not why?	Would you recommend involvement in this project/intervention to other professionals working with PwP and FC? If yes, why and if not why?

Participant responsiveness (Interaction with intervention; perceived usefulness, perceived meaningfulness, consultation frequency, help seeking behavior)

PwPD	FC	Coordinator/referring specialist
How did you experience your participation in the OPTIM-PARK project?<br>- Why did you decide to participate?<br>- What did you like about the intervention?<br>- What did you not like?<br>- Was it useful?<br>- What would you change?<br>- What would you advise researchers to do differently to make participation better?<br>- How did you experience contact with the coordinator?<br>- How did you experience contact with the researchers?<br>- What did you achieve from participating?<br>- What did you achieve as a couple?<br>- How has this affected everyday life with PD?	How did you experience your participation in the OPTIM-PARK project?<br>- Why did you decide to participate?<br>- What did you like?<br>- What did you not like?<br>- Was it useful?<br>- What would you change?<br>- What would you advise researchers to do differently to make participation better?<br>- How did you experience contact with the coordinator?<br>- How did you experience contact with the researchers?<br>- What did you achieve from participating?<br>- What did you achieve as a couple?<br>- How has this affected everyday life with PD?	How did you experience your participation in the OPTIM-PARK project?<br>- Why did you agree to participate?<br>- What did you like?<br>- What did you not like?<br>- Was it useful?<br>- What would you change?<br>- What would you advise researchers to do differently to make participation better?<br>- How did you experience contact with the PwP and FC?<br>- How did you experience contact with the researchers?

Delivery of intervention (Perceptions on delivery; content, consultation format, time spent)

PwPD	FC	Coordinator/referring specialist
Did the project fit your needs as you perceive them?<br>- Assessment of needs, use of ECPC or other tools<br>- What did you think of the tools used to address your needs<br>- Information about resources<br>- Referral to resources and services<br>- Time spent with coordinator	Did the project fit your needs as you perceive them?<br>- Assessment of needs, use of CSNAT or other tools<br>- Information about resources<br>- Referral to resources and services<br>- Time spent with coordinator<br>- What did you think of the tools used to address your needs<br>-	Were you able to provide the intervention as planned?<br>- Assessment of needs, use of tools (ECPC, CSNAT)<br>- Information about resources<br>- Referral to resources and services<br>- Time spent with PwPD and FC<br>- What did you think of the conversation tools that were used to assess participants needs?

How was the coordinator's ability to understand your specific situation and needs?<br><br>Were there any costs involved for you?<br><br>How has it been having a coordinator that you could contact?<br>- impact on everyday life?<br>- ability to cope with challenges related to PD?	How was the coordinator's ability to understand your specific situation and needs?<br><br>Were there any costs involved for you?<br><br>How has it been having a coordinator that you could contact?<br>- impact on everyday life?<br>- ability to cope with challenges related to PD?	In your opinion, did this project facilitate a more individualized approach in providing information about (and referrals to) resources and services as needed by PwPD and FC? If yes, why and if not, why?<br><br>Were you able to fit the work in your allocated hours?<br><br>Were any costs involved for you or your institution?

<u>Contextual factors</u> (Cross-national factors; community setting, Country specific; design of intervention aspects)

PwPD	FC	Coordinator/referring specialist
		In your opinion, is this initiative needed within the context of PD management in your community? If yes, why and if not, why?<br><br>Have you encountered any difficulties in the implementation of this intervention? If so, what?<br>- Any facilitators?<br>- What is needed for this intervention to be successfully implemented?

Acceptability of the outcome measures (Perception of the questions posed, did they find questions suitable? Administration: time spent, format (paper/digital), data collection procedures)

PwPD	FC	Coordinator/referring specialist
What did you think about the questionnaires you had to complete before and after participating in the intervention?<br>- Particularly easy/difficult/annoying questions?<br>- Time spent to complete, appropriate?<br>- Format for completion?<br>- Sufficient help to understand questions?<br>- Other..	What did you think about the questionnaires you had to complete before and after participating in the intervention?<br>Particularly easy/difficult/annoying questions?<br>- Time spent to complete, appropriate?<br>- Format for completion?<br>- Sufficient help to understand questions?<br>- Other..	

Closing questions for all groups:
Are there any changes that you think would make the project/intervention better?
Is there anything you expected us to ask you about that we didn't ask about?
Do you have any concluding remarks about your participation in the OPTIM-PARK project?
