# Supplementary material for: Feasibility and acceptability of the cross-national multisectoral OPTIM-PARK intervention for people affected with Parkinson’s disease and their family carers
Source: BMC Health Serv Res. 2026 Jun 23;26:999. doi: 10.1186/s12913-026-14912-5 (PMC13386678; doi:10.1186/s12913-026-14912-5)
Supplement: Supplementary file 3 — Supplementary Material 3 [file 12913_2026_14912_MOESM3_ESM.pdf]

II Template for analysis of the Logs and Mapping og Resources

Summary of individual mapping of resources and coordinator log

Country Norway

Participant ID 020101

1st meeting:
Choice of focus areas
- Understanding the disease
- Knowledge about food and medicine
- Handling emotions and worries
- Carer's mental well-being

2nd meeting:
Follow up on the focus areas
Carer's need for support – talk to a psychologist as a preventive measure
Job and economy, possibilities for working less and receiving economic compensation, receiving pension

3rd meeting:
Follow-up on possibilities for working less and receiving economic compensation
Conversation about prioritizing own resource and about self-care
Medical adjustments
Follow-up on talk about psychologist
Offer concerning psychologist's group sessions for caregivers

Supplementary information from the Log of resources
NOTE:<br>Seemingly, they have talked about a psychologist (seen in the minutes from the meetings) but not initiated this service
