# Supplementary material for: Feasibility and acceptability of the cross-national multisectoral OPTIM-PARK intervention for people affected with Parkinson’s disease and their family carers
Source: BMC Health Serv Res. 2026 Jun 23;26:999. doi: 10.1186/s12913-026-14912-5 (PMC13386678; doi:10.1186/s12913-026-14912-5)
Supplement: Supplementary file 4 — Supplementary Material 4 [file 12913_2026_14912_MOESM4_ESM.pdf]

Table 1 People with Parkinson's disease

Outcome measurement, mean (CI)	T0 Baseline n = 72		T1 Follow-up n = 58	
**Parkinson's Disease Questionnaire PDQ-39**				
Never, 0; Always, 4; Converted to index score 0–100 (Lower is better)				
Mobility	n = 69	50	n = 56	48
ADL	n = 72	42	n = 58	42
Emotional well-being	n = 71	38	n = 58	42
Stigma	n = 72	25	n = 58	25
Social support				
- For participants living with a partner (n = 56)		18		17
- For participants not living without a partner (n = 16)		18		19
Cognition	n = 72	38	n = 58	38
Communication	n = 71	8	n = 56	8
Bodily discomfort	n = 72	33	n = 55	33
**EQ-5D-5L**		n = 71		n = 57
No problem, 1; Extreme problem/unable, 5				
Mobility		2.6 (2.3–2.8)		2.6 (2.4–2.9)
Self-care		2.0 (1.7–2.3)		2.1 (1.8–2.4)
Activity		2.5 (2.2–2.7)		2.4 (2.0–2.6)
Pain		2.3 (2.1–2.6)		2.3 (2.0–2.5)
Anxiety		2.3 (2.0–2.5)		2.3 (1.9–2.6)
VAS 0–100 (Higher is better)	n = 69	57 (52–62)	n = 57	61 (55–66)
**Duke-UNC Functional Social Support Questionnaire**				
Not as much as desired, 1; As much as desired, 5				
1. I have people who care about what happens to me	n = 72	4.5 (4.2–4.7)	n = 58	4.6 (4.3–4.8)
2. I receive love and affection	n = 72	4.3 (4.0–4.6)	n = 57	4.5 (4.3–4.7)
3. I have chances to talk to someone about problems at work or with my housework	n = 69	4.1 (3.9–4.4)	n = 57	4.2 (3.9–4.5)
4. I have chances to talk to someone I trust about personal and family problems	n = 70	4.0 (3.8–4.4)	n = 58	4.2 (3.9–4.5)
5. I have chances to talk about financial matters	n = 69	4.3 (4.0–4.6)	n = 58	4.5 (4.2–4.7)
6. I receive invitations to go out and do things with other people	n = 70	3.9 (3.5–4.2)	n = 58	4.0 (3.8–4.4)
7. I receive useful advice about important things in life	n = 70	4.1 (3.9–4.4)	n = 58	4.3 (4.1–4.6)
8. I receive help when I am sick in bed	n = 69	4.2 (4.1–4.7)	n = 57	4.6 (4.4–4.8)

Table 2 Family carers

Outcome measurements, mean (CI)	T0 Baseline<br>n = 58	T1 Follow up<br>n = 47
**Parkinson's Disease Questionnaire PDQ-Carer-29**		
Never, 0; Always, 4; Converted to index score 0–100 (Lower is better)		
Social activity	n = 56 35	n = 46 35
Anxiety and depression	n = 55 33	n = 47 33
Self–care	n = 56 30	n = 47 30
Stress	n = 56 38	n = 46 42
**EQ-5D-5L**	n = 54	n = 45
No problem, 1; Extreme problem/unable, 5		
Mobility	1.2 (1.0–1.3)	1.2 (1.0–1.4)
Self–care	1.1 (1.0–1.2)	1.1 (1.0–1.3)
Activity	1.2 (1.1–1.4)	1.2 (1.1–1.4)
Pain	1.7 (1.4–1.9)	1.7 (1.4–2.0)
Anxiety	1.7 (1.4–2.0)	1.6 (1.3–1.9)
VAS 0–100 (Higher is better)	78 (73–83)	79 (74–83)
**Duke-UNC Functional Social Support Questionnaire**		
Not as much as desired, 1; As much as desired, 5		
1. I have people who care about what happens to me	n = 58 3.9 (3.6–4.3)	n = 46 4.2 (3.8–4.6)
2. I receive love and affection	n = 56 3.9 (3.4–3.2)	n = 46 4.3 (4.0–4.6)
3. I have chances to talk to someone about problems at work or with my housework	n = 55 4.0 (3.6–4.3)	n = 46 4.2 (3.8–4.5)
4. I have chances to talk to someone I trust about personal and family problems	n = 56 3.9 (3.5–4.2)	n = 46 4.1 (3.7–4.5)
5. I have chances to talk about financial matters	n = 56 4.0 (3.6–4.4)	n = 45 4.1 (3.7–4.6)
6. I receive invitations to go out and do things with other people	n = 56 3.7 (3.4–4.2)	n = 46 4.1 (3.7–4.5)
7. I receive useful advice about important things in life	n = 56 4.0 (3.6–4.3)	n = 45 4.1 (3.7–4.5)
8. I receive help when I am sick in bed	n = 55 3.9 (3.5–4.3)	n = 43 3.9 (3.4–4.4)
**Caregiver Burden**<br>*0-30 (Lower is better)*<br>Strongly agree (0) to strongly disagree (3) (cut-off: 20)	n = 52 11 (8.0–13)	n = 44 10 (7.0–12)
